# The Importance of Systematic Training in Operative Technics

**Published:** 1905-09-15

**Authors:** Fred. W. Gethro


### THE IMPORTANCE OF SYSTEMATIC TRAINING IN OPERATIVE TECHNICS.

BY FRED. W. GETHRO, D.D.S.

Read before the Southwestern Michigan Dental Society, April 12, 1905.

Following the request of your Secretary for something along the line of operative technics, I will endeavor to briefly outline what seems to me the more important points in the technic course as given in the school with which I am connected. This subject is necessarily a very broad one, for it embraces the foundation work for the entire field of operative dentistry.

It may be of interest to know how and why this course was added to the curriculum of the dental schools. Fifteen years ago no school in the country had an adequate technic course, but since then it has become one of the most important subjects of the curriculum.

Formerly it was expected that a student, before entering a dental school had served under a preceptor for a time varying from about six months to two years. This was considered essential to his successful school training. In European countries it is still considered the proper method to pursue. In this country we have long since arrived at the conclusion that it is not only unnecessary but in most cases it is detrimental to the best interest of the student. Ninety-five percent of the students that have served a term in a dental office come to the college with an extremely exaggerated idea of their preceptor's methods. These students start in college with many positive ideas as to just what they should do and what they should not do. To disabuse their minds of some of these false ideas and get them in a receptive mood, sometimes taxes the ingenuity and patience of a teacher.

I do not wish to be understood as casting reflections on any dentist who may have a student in his office, for there are many cases in which the student is benefited. It is rather a reflection of some serious difficulties which I have met in the handling of students.

The following is but an example of how a student's mind may be prejudiced. After a lecture on the treatment of pulpless teeth, one of the class said to me that he didn't see why it was necessary to use rubber dam and antiseptics, because he had been with a dentist two years and that dentist always filled root canals without the rubber dam, permitting the saliva to bathe the canals, claiming that the saliva was antiseptic. Another student when told he must sharpen the instrument he was cutting with, replied that he thought the instruments were sharpened when they were purchased, adding that he had never seen the dentist he was with sharpen an instrument. I believe that a student can get a great deal from a course with a good preceptor, but I believe that the best time for that training is after his graduation.

The aim of the technic course is to give the student the consecutive detail work of the technical procedures of operative dentistry as a preparation for actual operations in the mouth. I do not mean that we should stick exclusively to technical procedures, but rather appeal to the student from the practical. I want him to know the whys and wherefors. We must interest and enthuse him to get the best out of his efforts and to do that we must be able to show him the benefits to be gained in his after-practice.

The first work that the student does in the technic laboratory, after gaining a knowledge of dental nomenclature and studying the superficial parts of the teeth, is the dissection of each tooth. He is required to make from three to five dissections of every tooth in the mouth, some being made lengthwise, exposing the pulp chamber and root canal or canals in their entire length, and the others crosswise. This work familiarizes him with the several tissues of the teeth, namely the enamel cap, the dentine and the cementum, and permits of a study of the pulp chamber and root canals.

The proper technic course should give first the finger training, developing and strengthening the muscles of the fingers and acquiring a delicacy of manipulation. To demonstrate this point of development in the muscles of the fingers, I have brought with me the manudynamometer, an instrument designed by Dr. G. V. Black, to measure the force an operator exerts with the several instrument grasps. Sometimes during the clinic hours I want to have this instrument on exhibition, and I would like to have each member of the society try it. This ingenious instrument will tell you exactly the pounds pressure you are using. In some experiments with our present freshman class, I found on one occasion a student who could not exert over five pounds pressure. Unless that student develops strength rapidly, he had better quit the study of dentistry, for he is sure to be a failure, because this lack of finger power will seriously handicap him in his operating. While we have never conducted enough experiments to state definitely the advance made in finger strength, I do know that there is a vast improvement made during the technic course. The average pressure of our students when they enter is almost ten pounds. Experiments conducted in the junior class show an average of thirteen pounds. A very important point brought out in this technic training is the rest for one or more fingers so as to prevent any accident caused by the slipping of the instrument or the sudden movement on the part of the patient. It makes no difference whether one is using a cutting instrument, explorer, scaler, plugger, burnisher, engine or knife, a positive rest must be secured. The man who attempts to operate without using a rest, is sure, at some time, to do damage to his patient and himself.

There are two grasps used in most dental operations—one called the pen grasp, the instrument being held in the same manner as a pen, and the other the palm and thumb grasp. In this grasp the instrument is placed in the palm of the hand and held firmly by all the fingers excepting the thumb upon which it slides. In this case the thumb is also used as the rest finger.

But this alone is not enough to prevent slipping. One of the most important features is a sharp instrument. A sharp instrument is not likely to slip, a dull one is almost sure to slip. In the case of the sharp instrument little pressure is necessary to cause the instrument to cut effectively; with the dull instrument great pressure is necessary.

It is this combination of dull instrument, heavy pressure and no finger rest that causes fingers to be punctured, gum tissue to be lacerated aud in some cases parts of good tooth structure to be broken. How many cases do we have of torn rubber dam, caused from a cutting instrument slipping, a gold plugger slipping or a bur in a rapidly revolving engine running wild? These troublesome and sometimes disastrous accidents are not likely to occur with proper finger rests.

I feel that too much stress cannot be placed upon the sharpening of instruments. It is simply impossible for a man to become a skillful operator unless he can keep a keen edge on his cutting instruments. Dr. G. V. Black was once asked what obtundent he used for sensitive dentine, and he replied, "A sharp instrument." Never were truer words spoken for a sharp instrument will not only render the operation much less painful, but will shorten the time and do the work more effectively.

A good oil stone is a necessity. It should be of sufficient size and very hard. Many of our instruments are very small, and if we attempt to sharpen them on a soft stone, we will ruin both oil stone and instrument. The care of the oil stone is of great importance. The new stone should be soaked in oil and should be cleaned with oil on a cloth at the end of every day's use.

The study of instrumentation is a most important technic study. We use Dr. Black's set of forty-four cutting instruments, and in order to familiarize the students with these instruments—and the philosophy for making them—we require each student to make a full set in brass. By this process we not only familiarize him with the various forms but we find that many of our students have trained the eye so accurately, that at a glance at a given instrument, they can detect a variation in the width of the blade one-tenth of a millimeter, which is about 1-250 of an inch.

We use the metric system exclusively in all our work during the entire college course. Each student is required to have a steel millimeter gauge that will measure accurately to one-tenth of a millimeter. He uses this instrument constantly throughout his course. Instruments or burs are never spoken of as large, medium or small, each has a definite formula and this formula is used in all our teaching. This is necessary for accurate teaching. It is not possible for a teacher to tell a class to use a small hatchet or bur for a certain purpose, or a medium size spoon for another, and convey anything like an accurate impression. The carpenter would not speak of a small auger. He would say a 3-16 auger or a 5-8 auger. What seems small to one may seem large to another. I hope the time is not far off when the entire dental profession will adopt and use this metric system, for it will prove invaluable in our dental societies. Our papers and discussions will be much more accurate and valuable.

The blade of every cutting instrument has three measurements—the first is the width at the cutting edge and is given in tenths of a millimeter, the second is the length of the blade and is given in millimeters, and the third is the angle of the blade with the shaft or handle and is given in centigrade or hundreds of a circle. When the student is told to cut a groove in a cavity with hoe 6-2-6, he can make no mistake as to the size of the instrument to be used or the width of the groove to be made. If he is told to make a pit with an inverted cone bur 5-10 of a millimeter, measurements for burs always referring to the diameter, he knows exactly the size of the bur to use.

Every student carves in ivory six teeth, duplicating six typical models selected from extracted teeth. We require that he duplicate, the root portion with the same accuracy that he duplicates the crown. No carving is accepted unless accompanied by the natural tooth used as a model for means of comparison. This carving is valuable in proportion to the accuracy with which it is executed. This work serves the double purpose of giving him technical skill with his cutting instruments and teaching him external dental anatomy by a method more effective than any other. The carving of teeth is undoubtedly the most difficult technical work required of the student during his entire course, but the result is well worth the effort. The instruments used for carving are the same set of forty-four cutting instruments that he uses for all his technic work in his freshman year, and for his operating in his junior and senior years, and in his after practice.

Let me give you an illustration of the benefits of this carving: After the student has carved a mesio-buccal root of an upper first molar, he is well aware that as it leaves the crown portion it inclines to the mesial and buccal and then to the distal. Note of what assistance it is to him when he comes to treat this same root in the mouth. He has definite knowledge of just how his broach should be bent to correspond with the various inclinations of the root. By no other method with which I am familiar could he gain such exact knowledge of these minute details. The effort in this carving is to develop the idea of contour of the teeth. How often do we see gold fillings in the anterior part of the mouth that are conspicuous and offend our ideas of the esthetic? In many of these cases, had the fillings been properly contoured, the result would have been much less conspicuous and in some cases hardly noticeable. Harmony of contour does much to overcome lack of harmony of color. The same is true of porcelain or any other material used to reproduce lost tooth structure. If we are to replace teeth or parts of teeth and conceal our art, we must gain a minute detail knowledge of dental anatomy. One could write pages on the many benefits gained from this carving.

### TREATMENT OF TEETH.

I do not believe that any subject in operative dentistry needs more careful study and conscientious technical study than the treatment of teeth. I am of the opinion that the profession, taken as a whole, is not obtaining as good results as it should.

There are several reasons for this, and possibly the one that appeals with equal force to both the patient and operator is the financial part of it.

In our dental colleges we do not make a charge for the student's work time. This in itself is right. We only make charges where material is used. I believe that this is the starting point of this wrong idea. The student gains the impression that treatments bring him little or no remuneration, and that it is the filling or crown where he expects to be financially rewarded. Again the average patient does not expect to pay much for the treatment of a tooth, if the same dentist places the filling or crown.

We are to blame for this condition, for we have educated the people into this way of thinking. A charge should be made for every treatment and the patient should be made to understand that he is paying for each appointment, regardless of what we may do. A dentist should not make the same charge for a crown where the tooth has been treated and root filled that he would for one where he has to treat the tooth. A separate charge should be made for each treatment.

We endeavor to impress our students with the fact that to successfully treat teeth requires a higher development of technic than the successful insertion of fillings or the placing of crowns.

With few exceptions, regardless of whether we may have an exposed pulp with any of its attending manifestations, dead pulp, putrescent pulp, abscess or what not, the first thing that we require is that the cavity be prepared. This is necessary, first, because it gives better access, second, it places the tooth in the proper condition to receive temporary filling and third, removes any remaining decay or infected tissue. Following this the field of operation should be rendered antiseptic by the use of a potent antiseptic. How many times do we find cases where root canals are being treated with more or less infected material left in the cavity. Every time the broach or any other instrument is placed in the canal, the operator is likely to carry more infection to it. There may be a few cases where one could not well prepare the cavity and remove all the decayed dentine, such as cases where we have a severe pulpitis or a very sore tooth, and yet in many of the former we can generally apply some soothing medicine to allay the pain, and in the latter we can, by various methods, support the teeth so that we may work on them with comparatively little pain to the patient. After the cavity is prepared we are ready to remove the pulp. This is effected by means of the barbed broach. Many of the small, and canals which sometimes seem impossible to thoroughly clean out, can, by the use of suitable sized barbed broaches, be enlarged and made accessible. The barbed broach should never be twisted, but should be placed in the canal as far as it will go and then withdrawn. In the withdrawing, the broach acts as a flexible file, and each barb will cut away the more prominent parts, enlarging and straightening the root canal. This process should be continued until the canal is sufficiently enlarged to make it accessible. This can be best taught in the technic course on teeth out of the mouth.

After the canal is thoroughly cleaned and dried it is ready for filling. The walls of the canal should be moistened with eucalyptol. This acts as a solvent for the gutta percha that is to follow. We impress on the student that he use chloro percha sparingly, only using it in small root canals. In these small root canals a limited amount is worked in on a broach wound with cotton. The root canal plugger has been selected previously and a gutta percha cone fastened to its point by slightly warming the end of the plugger point. We must use a plugger point of sufficient size, so that it will pack the filling material and not simply puncture it. As an object lesson in root filling we have every student file away the mesial and buccal root canals of upper first molars, and the mesial roots of a lower first molar in which the canals have been filled previously. This filling exposes the root canal in its entire length. It is unnecessary to add that in some cases we find imperfectly filled canals and occasionally a piece of broken broach is discovered. I believe this optical demonstration causes the student to think more seriously about this subject of root filling.

At this time we take up the study of cavity preparation and at the same time begin to prepare the cavities in natural extracted teeth, bone and in carved ivory teeth.

In this technic course we are not teaching the student the pathology of decay, but we are teaching him to observe the physical characteristics of decay.

Great stress is placed on the instrumentation of cavity preparation. The aim is to teach the student to think in instrument form. This is the great time saver in the preparation of cavities. Few instruments are necessary for the preparation of a given cavity.

To demonstrate the instrumentation of cavity preparation, we use a number of large models of teeth with a set of instruments which are duplicates of the cutting instruments used by the students. These instruments are made on a scale of fifteen diameters and can be readily seen from any part of the lecture room. All cavity preparation must be carried through with a definite order of procedure. Each step is completed before progressing to the next. The first step is outline form and includes the preparation of the walls of the entire outline of the cavity. The second step is resistance form and consists mainly of a flat wall or base and is usually at right angles to the stress of the filling. The third step is retention form. The fourth step is convenience form. The fifth step is to remove any decayed dentin. The sixth step is to smooth the walls, bevel the cavo-surface angle and make the final preparation, or toilet the cavity.

This order of cavity preparation, as well as other technic teaching is begun in the freshman year and continued in the junior and senior years. It is this continued uniformity of teaching that eventually teaches the student to do things with a definite system.

The various filling materials are studied and fillings are placed in natural teeth, in bone and in carved teeth.

The work of each student is mounted in a specially designed box and is handed in a number of times during the year for inspection and at the end of the year for final grading. All of the work is kept on exhibition two years in our library and museum or until the week of graduation, when it is returned to the owner.

